# Effects of testosterone enanthate on aggression, risk-taking, competition, mood, and other cognitive domains during 28 days of severe energy deprivation

**DOI:** 10.1007/s00213-023-06502-8

**Published:** 2023-12-01

**Authors:** Harris R. Lieberman, John A. Caldwell, Oshin Vartanian, Owen T. Carmichael, J. Philip Karl, Claire E. Berryman, Kishore M. Gadde, Philip J. Niro, Melissa N. Harris, Jennifer C. Rood, Stefan M. Pasiakos

**Affiliations:** 1 Military Nutrition Division, US Army Research Institute of Environmental Medicine (USARIEM), Natick, MA 01760-5007 USA; 2 University of Toronto, Toronto, ON Canada; 3 Laulima Government Solutions, Frederick, MD USA; 4 Defence Research and Development Canada, Toronto, ON Canada; 5 Louisiana State University’s Pennington Biomedical Research Center, Baton Rouge, LA USA; 6 Department of Surgery, University of California Irvine, Orange, CA USA; 7 Office of Dietary Supplements, National Institutes of Health, Bethesda, MD USA

**Keywords:** Fatigue, Food deprivation, Body composition, Hormones, Memory, Executive function, Energy restriction

## Abstract

**Rationale:**

Behavioral effects of testosterone depend on dose, acute versus sustained formulation, duration of administration, personality, genetics, and endogenous levels of testosterone. There are also considerable differences between effects of endogenous and exogenous testosterone.

**Objectives:**

This study was the secondary behavioral arm of a registered clinical trial designed to determine if testosterone protects against loss of lean body mass and lower-body muscle function induced by a severe energy deficit typical of sustained military operations.

**Methods:**

Behavioral effects of repeated doses of testosterone on healthy young men whose testosterone was reduced by severe energy deficit were examined. This was a double-blind, placebo-controlled, between-group study. Effects of four weekly intramuscular injections of testosterone enanthate (200 mg/week, *N* = 24) or matching placebo (*N* = 26) were evaluated. Determination of sample size was based on changes in lean body mass. Tasks assessing aggression, risk-taking, competition, social cognition, vigilance, memory, executive function, and mood were repeatedly administered.

**Results:**

During a period of artificially induced, low testosterone levels, consistent behavioral effects of administration of exogenous testosterone were not observed.

**Conclusions:**

Exogeneous testosterone enanthate (200 mg/week) during severe energy restriction did not reliably alter the measures of cognition. Study limitations include the relatively small sample size compared to many studies of acute testosterone administration. The findings are specific to healthy males experiencing severe energy deficit and should not be generalized to effects of other doses, formulations, or acute administration of endogenous testosterone or studies conducted with larger samples using tests of cognitive function designed to detect specific effects of testosterone.

## Introduction

This study evaluated the effects of testosterone on cognition and mood. It was the secondary arm of a clinical trial of young healthy men designed to determine whether exogenous testosterone administration is an effective countermeasure for the adverse effects of severe sustained energy deficit induced by substantial dietary energy restriction and extensive physical exercise simulating military operations (Pasiakos et al. 2019). Energy intake during military operations is often inadequate due to extensive sustained physical activity and undernutrition (Nindl et al. 2002; Tharion et al. 2005). In soldiers engaged in stressful military operations, substantial energy deficits result in low levels of endogenous testosterone (Moore et al. 1992). It is well-established that administration of testosterone to hypogonadal men increases muscle mass and strength (Varanoske et al. 2022).

Testosterone is currently approved by the FDA for treating men with low testosterone levels due to primary hypogonadism or hypogonadotropic hypogonadism (Nguyen et al. 2015). Its beneficial effects on body composition are well documented in this population (Wang et al. 2000; Zając et al. 2014; Corona et al. 2016). In addition, studies have examined the relationship between endogenous testosterone levels and a wide-variety of cognitive functions in humans, including spatial ability, aggression, memory, executive function, mood, and risk-taking, sometimes with conflicting results, and that do not always agree with studies of other animals or popular perception of the behavioral effects of testosterone such as its effects on aggressive behavior (Shute et al. 1983; Christiansen and Knussmann 1987; Moffat and Hampson 1996; Morley et al. 1997; Silverman et al. 1999; Pope et al. 2000; Muller et al. 2005; Beauchet 2006; Johnson and Breedlove 2010; Geniole et al. 2019). Several studies have assessed the impact of exogenous testosterone administration on human behavior, and the findings of these can be contradictory as noted in several recent reviews, perhaps due in part to differences in test populations, dose, duration, sex, personality, genetics, and formulation of testosterone. There are also considerable differences in the behavioral tasks used and the number of subjects tested in such studies (O’Connor et al. 2002, 2004; Dreher et al. 2016; Elliott et al. 2017; Yalamanchi and Dobs 2017; Ponce et al. 2018; Buskbjerg et al. 2019; Geniole et al. 2019; Diem et al. 2020).

### Cognition

The results of the studies evaluating the effects of exogenous testosterone administration on cognitive function vary substantially. Studies of acute vs. chronic administration of testosterone often assess different behavioral functions since they are conducted for different purposes. Studies with clinical populations or with formulations of testosterone designed for chronic administration often use cognitive test batteries that assess multiple behavioral functions (Cherrier et al. 2001, 2015; Lašaitė et al. 2017; Kenny et al. 2004; Vaughan et al. 2007; Resnick et al. 2017). Studies designed to detect effects of testosterone on specific aspects of cognitive function, such as aggressive behavior, often examine effects on single highly specialized tasks designed for such studies (Bird et al. 2019; Dreher et al. 2016; Geniole et al. 2019; Nave et al. 2017; Knight et al. 2022; Kutlikova et al. 2023; Losecaat Vermeer et al. 2020).

In a study of chronic effects of testosterone, Cherrier et al. (2001, 2015) found that in normal men, 6 weeks of treatment with 100 mg injections of testosterone enanthate (a sustained-release formulation) improved spatial memory, spatial ability, and verbal memory. Lašaitė et al. (2017) reported that men diagnosed with hypogonadotropic hypogonadism who received intramuscular testosterone undecanoate injections (1000 mg; another sustained-release formulation) every 10–14 weeks for 2 years experienced improvements in attention, visual scanning ability, executive functions, and psychomotor speed. However, a study by Kenny et al. (2004) failed to find significant improvements in the cognitive performance of men with low testosterone, who were experiencing early cognitive decline, when they were treated with 200 mg testosterone enanthate every 2 weeks for 1 year. Vaughan et al. (2007), found that healthy older men with low serum testosterone and no evidence of cognitive impairment improved on a test of attention, but not on tests of executive functions, visuospatial skills, or visual and verbal memory skills following 200 mg of intramuscular injections of testosterone enanthate every 2 weeks for a period of up to 3 years. These findings are consistent with those of Resnick et al. (2017) who reported that 1 year of testosterone treatment (with testosterone gel, 1% concentration) of older men with low testosterone and age-associated memory impairment was not associated with improved memory or other cognitive functions. These conflicting results are consistent with recent meta-analyses concluding the available evidence that chronic administration of exogenous testosterone improved cognition or overall well-being was inconclusive (Elliott et al. 2017; Yalamanchi and Dobs 2017; Ponce et al. 2018; Buskbjerg et al. 2019; Diem et al. 2020).

### Mood

Several investigations have assessed the effects of exogenous testosterone on mood state, and the results have been conflicting (Elliott et al. 2017; Diem et al. 2020). Using a daily mood diary, Anderson et al. (1999) found testosterone enanthate (200 mg per 3 weeks) in hypogonadal men resulted in more positive mood states, but O’Connor et al. (2004) reported testosterone undecanoate (single 1000 mg dose) increased anger and hostility as assessed by the Profile of Mood States (POMS) in eugonadal men. In contrast, Salmimies et al. (1982) found no change in mood state in hypogonadal men as measured by a mood scale after administration of either 100 or 400 mg doses of testosterone enanthate every 4 weeks for a total of 5 months.

### Collaboration and generosity

Testosterone administration has been reported to either disrupt or improve collaborative behavior and/or generosity. Zak et al. (2009) observed that artificially elevating testosterone via acute topical application of a non-sustained release (1% testosterone gel) made healthy men 27% less generous towards strangers and generally more antisocial. A large study of males (*N* = 400) of the acute effects of testosterone on cooperation found that individual complex personality differences interacted with the effects of testosterone on tasks requiring cooperation (Bird et al. 2019). In a study of the acute effects of testosterone on males (*N* = 187), its administration decreased prosocial behavior when individuals were monitored by an audience (Kutlikova et al. 2023). In another study of acute administration of testosterone to males (*N* = 115) on competitive tasks, the effects of testosterone interacted with individual levels of the stress hormone cortisol (Knight et al. 2022).

### Risk-taking

Conclusions about the relationship between testosterone and risk-taking seem to depend largely on whether endogenous levels of testosterone or exogenous administration are studied. Men with high endogenous testosterone levels appear to make riskier choices than those with lower levels (Apicella et al. 2008; Stanton et al. 2011; Ristvedt et al. 2012); although there are exceptions (see Apicella et al. 2015). The effects of exogenous testosterone on risk-taking behavior are not clear. Administration of an aromatase inhibitor (letrozole 2.5 mg/day) which artificially increases testosterone levels of healthy men increased risk-taking, particularly under conditions of unknown payoff probabilities (Goudriaan et al. 2010).

### Aggression

Many studies of animals including non-human primates, correlational research with humans, and anecdotal evidence suggest that testosterone alters aggressive behavior, especially in males. A review of the effects of supraphysiological doses versus therapeutic doses of exogenous testosterone on aggression cited some evidence supraphysiological doses were associated with increased aggression and anger (O’Connor 2007). A dose–response investigation by Su et al. (1993) found that administering non-sustained-release testosterone in high doses over 3 days (i.e., high-dose [240 mg/day] vs. low-dose [40 mg/day] methyltestosterone) caused a significant but subtle increase in irritability, violent feelings, and hostility in healthy men. Another study reported that chronic, supraphysiological doses of testosterone, 200 mg of testosterone enanthate given weekly for 8 weeks, did not increase aggression or mood disturbance (O’Connor et al. 2002).

Nave et al. (2017) who conducted a study of acute high-dose testosterone administration to 243 men found its administration-reduced cognitive reflection in a test of this aggression-related parameter. In another study of acute administration of testosterone, males (*N* = 40) treated with testosterone were more likely to punish adversaries in a modified version of the Ultimatum Game, considered to be a measure of aggression (Dreher et al. 2016). Geniole et al. (2019) studied the effects of acute, high-dose testosterone administration in a large sample of men (*N* = 308) and found that personality and a specific genetic factor was associated with increased aggression in a competitive task. A recent comprehensive meta-analysis by Geniole et al. (2020) found that the literature did not provide conclusive evidence of a causal association of testosterone with aggression. However, another recent meta-analysis of 12 randomized controlled trials with healthy men found a small increase in self-reported aggression following testosterone administration (Chegeni et al. 2021). It appears that the effects of exogenous testosterone on aggression are much more complex than originally thought and are moderated by a variety of factors (Archer et al. 2005; Geniole et al. 2020). For example, a study that administered acute testosterone to 113 males found personality and genetic differences interacted with the effects of testosterone and that testosterone increases status-related motivation (Losecaat Vermeer et al. 2020). Wagels et al. (2018) reported that effects of testosterone are dependent on situational factors and “testosterone reinforces the conditional adjustment of aggressive behavior but not aggressive behavior per se”.

### Summary

With regard to cognition, there are reports of positive effects on spatial ability, verbal memory, and feelings of fatigue (O’Connor et al. 2004; Beauchet 2006) and reports of negative effects on anger-hostility, mania, aggression, egocentricity, selfishness, skepticism, and on some types of risk-taking tasks. However, these findings have not been consistently replicated by other studies (Pope et al. 2000; O’Connor et al. 2004; Zak et al. 2009; Johnson and Breedlove 2010; Goudriaan et al. 2010; Wright et al. 2012). It is difficult to draw firm conclusions regarding the effects of exogenous testosterone for a variety of reasons including the wide range of doses used, the different formulations of testosterone used (time-release vs. acute), the age groups tested, genetic and personality differences, the genders under investigation, and differences in cognitive tests used.

## Objective

The primary arm of this study determined if chronic administration of sustained release testosterone prevented the degradation in muscle mass and strength that occurs during sustained military operations, and it found that testosterone increased muscle mass but did not affect the physiological performance of volunteers (Pasiakos et al. 2019). There were a number of secondary findings associated with the conduct of this clinical trial in addition to the primary report and this paper (Pasiakos et al. 2019). In several reports, the effects of testosterone on androgen receptor expression, protein synthesis and the proteome were examined (Howard et al. 2020, 2022). Furthermore, in a series of reports, the metabolic consequences of testosterone administration were studied including its effects on iron availability for erythropoiesis (Hennigar et al. 2020), ghrelin and appetite (Karl et al. 2020), metabolomics (Stein et al. 2022), and metabolic and substrate oxidation (Margolis et al. 2023). Several papers based on functional magnetic resonance imaging (fMRI) and cognitive measures other than those reported on here have also been published and are discussed below when appropriate (Carmichael et al. 2021; Vartanian et al. 2023).

This study provided a unique opportunity to investigate the effects of 4 weeks of testosterone administration on the behavior of healthy men whose testosterone levels were artificially reduced by severe energy deficiency. If testosterone is used in military and/or civilian populations, it is essential to determine its effects on cognitive function and mood since many have been reported to be sensitive to endogenous testosterone levels or exogenous testosterone. In addition, since volunteers were followed for several weeks after testosterone treatment was discontinued, any resulting withdrawal effects could be observed. This arm of the study was designed to address the hypothesis that adverse changes in mood and cognition would be observed in placebo-treated, energy-deficient volunteers, and these effects would be attenuated by testosterone administration. Additional information regarding volunteer recruitment, study design, assessment of physical performance, and nutritional and biochemical function of this clinical trial has been reported elsewhere (Pasiakos et al. 2019; Hennigar et al. 2020; Howard et al. 2020; 2022; Karl et al. 2020; Carmichael et al. 2021; Margolis et al. 2023; Stein et al. 2022; Vartanian et al. 2023).

## Materials and methods

### Volunteers

Healthy, drug-free, normal weight, physically active (≥ 2 days/week aerobic and/or resistance exercise) men aged 18–39 years, with normal total testosterone concentrations (300–1001 ng/dL), were recruited from the Baton Rouge, LA community. Volunteers with prostate-specific antigen concentrations > 3 ng/mL, hematocrit > 50%, a positive urine drug screen, or reported use of anabolic steroids, human growth hormone, or nutritional testosterone precursor-like supplements within the past 6 months were excluded. In addition, subjects taking any medications or unwilling to refrain from all medication use 4 weeks prior to and throughout the entire study period, as well as those unwilling to refrain from alcohol, smoking (any nicotine product), caffeine use, and/or dietary supplement use throughout the entire study period, were excluded from participation. Additional inclusion and exclusion criteria are provided elsewhere (Pasiakos et al. 2017). This study was approved by the Pennington Biomedical Research Centre Institutional Review Board and the Human Research Protection Office of the US Army Medical Research and Materiel Command. Volunteers provided written informed consent. This study was conducted as part of a registered clinical trial (ClinicalTrials.gov; NCT02734238).

### Overall design

This was a randomized, double-blind, placebo-controlled registered clinical trial consisting of three consecutive phases in which 24 volunteers were randomly assigned to the testosterone group (average age = 25; weight = 81 kg; and BMI = 25 kg/m^2^) and 26 others were randomly assigned to the placebo group (average age = 25; weight = 77 kg; and BMI = 24 kg/m^2^). The study was powered at the 0.90 level based on detecting a change in lean body mass not the biochemical measures used.

Phase 1 was a 14-day (days 1–14), free-living, eucaloric diet period. During this phase, total daily energy expenditure for the diets provided was determined using the Mifflin St. Jeor Equation based on an activity factor of 1.3 to account for activities of daily living, in combination with results from 7-day accelerometer data and 3-day activity logs collected during screening visits (Mifflin et al. 1990). Mean relative intake for phase 1 was 34.1 ± 5.0 kcal/kg/day with 20% of total daily energy requirements derived from protein, 30% derived from fat, and 50% derived from carbohydrate. Examples of foods provided during phase 1 and phase 2 are egg white omelets, deli turkey pita pockets, chicken pesto pasta, and trail mix. Volunteers maintained their habitual physical activity levels during phase 1. Diet and physical activity adherence were verified by research dietitians, accelerometry, and by ensuring body mass was maintained within ± 2%. Neither energy expenditure nor dietary intake differed between the testosterone and placebo groups.

After day 14, volunteers were admitted to an inpatient unit where they underwent a 28-day (phase 2; days 15–42), highly controlled, individualized exercise- and diet-induced energy deficit designed and monitored to ensure they maintained daily energy expenditure of 55% of their baseline level. During this phase, volunteers resided in small groups. The 55% energy deficit was accomplished by increasing exercise to elevate total daily energy expenditure by 50% above phase 1 levels and restricting energy intake for each volunteer to 45% of this amount. Mean relative intake for phase 2 was 24.1 ± 3.9 kcal/kg/day with 28% of total daily energy requirements derived from protein, 30% from fat, and 42% from carbohydrate. All meals were provided, and they were consumed under investigator supervision. To increase phase 2 total daily energy expenditure, individualized exercise consisted of varied, low-, moderate-, and high-intensity (40–85% of predetermined peak rate of oxygen uptake) aerobic-type exercise, including treadmill and/or outdoor walking and/or running, elliptical, stationary bike, and weighted backpack (carrying 30% of body mass) walking. Exercise sessions were conducted at 0600, 1100, and 1600, with an optional session available at 1900. On average, volunteers performed 3.5 exercise sessions per day, all directly supervised by research staff. The physical activity prescription was verified biweekly and adjusted as needed to achieve the desired energy expenditure using open circuit indirect calorimetry. Upon completion of phase 2, volunteers were released from the inpatient unit and instructed to return to their pre-study, ad libitum, habitual diet, and physical activity routines. Mean relative intake for phase 3 was 43.0 ± 20.3 kcal/kg/day with 15% of total daily energy requirements derived from protein, 40% derived from fat, and 45% derived from carbohydrate.

### Testosterone/placebo dosing

**Fig. 1:**
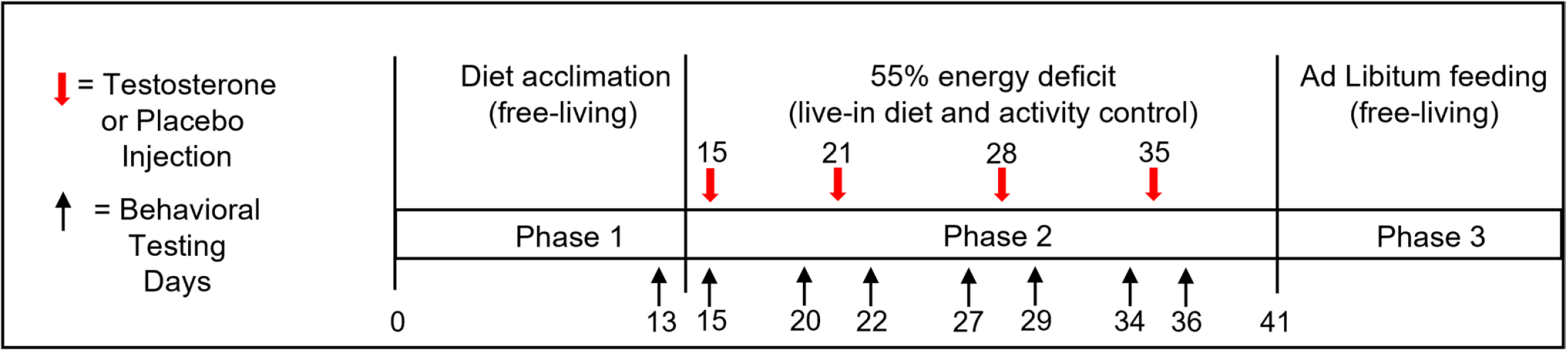
Overview of the study schedule showing the 3 phases of the study: diet acclimatization/free-living, energy deficit/live-in, and ad libitum feeding/free-living. Mood and cognitive test days (days 13, 15, 20, 22, 27, 29, 34, 36, and 41) are noted across the bottom with black arrows, and testosterone/placebo injection days (days 15, 21, 28, and 35) are indicated by red arrows across the top

At the start of phase 2 (day 15), a software-generated plan (SAS Institute, Cary, NC, USA, version 9.4) was used to randomize volunteers to the testosterone or placebo condition. The testosterone group (*N* = 24) received 4 intramuscular injections of testosterone enanthate (200 mg of testosterone/week) in sesame oil. The placebo group (*N* = 26) received 4 doses of sesame oil alone (1 mL sesame oil/week). Testosterone or placebo was administered at 0700 on days 15, 21, 28, and 35 (Fig. 1). The dose was selected to maintain near normal testosterone plasma levels despite the imposed severe energy deficit which was expected to lower endogenous testosterone levels of placebo-treated volunteers. Study volunteers and study staff were blinded to group assignment until data collection was complete and initial data analyses were conducted.

### Testing schedule

Several training sessions to familiarize volunteers with the cognitive and mood measures were conducted prior to baseline administration of cognitive and mood tests on day 13, 1 day prior to the end of phase 1 (Fig. 1). Following the baseline test session, cognitive/mood evaluations were conducted on day 15 (the day the first injection was given; approximately 4 h post-injection), day 20 (1 day prior to the second injection), day 22 (1 day after the second injection), day 27 (1 day prior to the third injection), day 29 (1 day after the third injection), day 34 (1 day prior to the fourth injection), day 36 (1 day after the fourth injection), and day 41 (5 days after the last injection and 1 day prior to the end of the energy-deficit phase of the study; Fig. 1).

During the final phase of the investigation, volunteers were released into their free-living environment and returned to their pre-study dietary and activity routines. During this phase volunteers were tested at the midpoint (day 54) and at the endpoint which varied somewhat because it was based on the time required for a volunteer’s body mass to recover to ± 2.5% of their initial body mass (approximately day 85).

### Cognitive function and mood state assessment

A battery of cognitive tasks was used to evaluate the effects of energy deficit and exogenous testosterone administration on a variety of cognitive functions reported to be affected by testosterone administration (Yalamanchi and Dobs 2017) or sensitive to the effects of psychotropic drugs or other independent variables (Archer et al. 1998; Lieberman et al. 2002; O’Connor et al. 2002; Goudriaan et al. 2010). Functions investigated are described below and included fatigue and other aspects of mood such as anxiety and depression; aggressive thoughts; risk-taking propensity; perception of other’s feelings and intentions (social cognition); and vigilance, memory, reaction time, and executive function. The sequence of test administration within all test sessions did not vary. The test battery was administered between 0900 and 1400 h (depending on the phase of the study) and required approximately 90 min to complete.

#### Buss–Perry Aggression Questionnaire

This test has been used to examine the effect of testosterone on aggressive impulses (O’Connor et al. 2004). It is a 29-item questionnaire that assesses aggressive thought patterns. Volunteers rank statements on a 5-point continuum from “extremely uncharacteristic of me” to “extremely characteristic of me” (Buss and Perry 1992). Examples of items include “Once in a while I can’t control the urge to strike another person,” “Given enough provocation, I may hit another person,” and “If somebody hits me, I hit back.” The results are provided as scores on 4 scales: Physical Aggression, Verbal Aggression, Anger, and Hostility. The total score for aggression is the sum of these scale scores and ranges from 29 to 145. Higher scores indicate higher aggressive behavior (the average for males is 77.8). There is a strong correspondence between pencil-and-paper measures of aggression and their behavioral counterparts (Carlson et al. 1989).

#### Profile of Mood States (POMS) Questionnaire

This is a 65-item inventory of mood states sensitive to a wide variety of drugs and stressors (McNair et al. 1971; Banderet and Lieberman 1989; Lieberman et al. 2002). Volunteers rate each of 65 mood-related adjectives on a five-point scale, in response to the question, “How are you feeling right now?” The adjectives factor into six mood sub-scales (Tension/Anxiety, Depression/Dejection, Anger/Hostility, Vigor/Activity, Fatigue/Inertia, and Confusion/Bewilderment) as well as a Total Mood Disturbance score which is calculated by adding 5 sub-scales (Tension/Anxiety, Depression/Dejection, Anger/Hostility, Fatigue/Inertia, and Confusion/Bewilderment) and subtracting sub-scale Vigor/Activity.

#### Balloon Analogue Risk Task (BART)

Since risk-taking behavior is reported to be altered by testosterone administration (Goudriaan et al. 2010), this computer-administered task was given to assesses willingness to take risks versus “playing it safe.” To complete it, volunteers repeatedly fill simulated balloons with air (Lejuez et al. 2002). Points are given to volunteers for delivering a simulated puff of air and increasing the volume of the balloon. Volunteers were informed that there was no actual financial outcome related to the points earned during this task. The more the balloon is expanded, the more points earned. However, all points are lost if the balloon is “over-inflated” and pops. In this study, simulated balloons that randomly popped after either 8, 32, or 128 puffs were tested, and scores were number of balloons popped in each category during a session.

#### Ultimatum Game

The Ultimatum Game is used to examine the association of testosterone levels with aggressiveness versus cooperation (Burnham 2007). It assesses negotiation and cooperation and appears to the volunteer to be a competition with another individual, but in fact, a computer simulates the other individual. The results often conflict with what would be expected based on rational economic theory. In this game, it appears to the subject that two players are being offered a chance to win a sum of money and instructed they must divide it. The proposer suggests how to split the sum, and the responder either accepts or rejects the deal. If the deal is rejected, neither player gets anything. The optimal rational solution, according to game theory, is for the proposer to offer the smallest possible share and for the responder to accept it; however, the most frequent outcome is a fair share. Low proposals are often rejected by responders, perhaps due to the fact that subjects may wish to manage their reputations to demonstrate, they will not accept unfair treatment. Data on offers accepted and rejected, time outs, and reaction time were collected. There were 10 offers per trial, with low offers ranging from $2 to $11 and high offers ranging from $20 to $24. Volunteers were informed that there was no actual financial outcome and no money would be earned while completing this task.

#### Reading the Mind in the Eyes Test

This test examines ability to attribute or infer beliefs, intentions, and desires of others (Baron-Cohen et al. 2001). It is used to assess social cognition in individuals with autism spectrum disorders and schizophrenia as well as normal adults (Prevost et al. 2014). It was used to examine effects of testosterone since levels are reported to be associated with aggression, selfishness, and suspicion about intentions of others (Christiansen and Knussmann 1987; Johnson and Breedlove 2010). Volunteers are presented with 36 pictures of a person’s eyes and adjacent facial area, along with four descriptive words. They then choose the word that best describes the emotional or mental state of the person in the image. Outcome variables are correct responses, incorrect responses, time outs, and reaction times.

#### Scanning Visual Vigilance Task

This task is sensitive to low doses of hypnotic drugs and stimulants, nutritional factors, environmental conditions, and sleep loss (Fine et al. 1994; Lieberman et al. 1998). The volunteer is required to continuously scan a computer screen to detect the occurrence of an infrequent, difficult to detect stimulus presented on average once a minute. Upon detection of the stimulus, the volunteer responds as rapidly as possible. Correct responses, false alarms, and reaction times are reported.

#### Psychomotor Vigilance Test (PVT)

This is a 10-min test of simple visual reaction time widely used to assess alertness (Dinges and Powell 1985). The test requires volunteers to sustain attention and respond to stimuli presented at random intervals on a computer screen by pressing a button. Parameters recorded include reaction time, premature responses, correct responses, and number of lapses (which are long duration greater than 500 ms or “time-out” responses).

#### Matching to Sample

This test assesses short-term spatial memory (working memory) and pattern recognition (Thomas et al. 1989; Lieberman et al. 2002). Volunteers are presented with a series of 8 × 8 and 16 × 16 matrices on a color screen for 4 s. After each matrix is presented, the screen is blanked for 8 s or 16 s after which two matrices are shown: the original matrix and a second slightly different matrix. The volunteer must select the correct comparison matrix within 15 s. Time-out errors, correct responses, and reaction times are assessed.

#### Grammatical Reasoning test

This test assesses language-based logical reasoning and is frequently used to study the effects of various treatments on verbal reasoning (Baddeley 1968). On each trial, the letters AB or BA are presented following a statement such as “A precedes B.” The volunteer must report whether or not each statement correctly describes the order of the two letters. A session lasts for 32 trials and is scored for accuracy and reaction time.

#### N-back task

This task requires on-line monitoring, updating, and manipulation of working memory (Soveri et al. 2017). Volunteers are asked to monitor the presentation and order of a series of letters and to indicate when the currently presented letters are the same as the one presented “*n*” trials back (in this study either 1-back or 2-back). Dependent measures include response times and three measures of accuracy (overall correct, correct *n*-back, and false positives).

### Data analysis

#### Testosterone assay analysis

Blood samples for testosterone assays were collected on baseline day [day 0], days 14, 28, 42, and 56, and the end-of-study day. Testosterone levels (from blood assays) were analyzed in a 2-way, mixed factorial ANOVA for “drug” which consisted of 2 levels (testosterone versus placebo) and “day” which consisted of 6 levels—each day a blood sample was assayed for testosterone. Days when testosterone assays were conducted did not always correspond to the days it, or placebo, was administered (Fig. 1). Degrees of freedom were corrected using the Greenhouse–Geisser procedure to adjust for potential sphericity violations across the repeated-measures factor (day). Since one group of subjects received testosterone injections during energy-deficit days while the other group received a placebo on these days, a significant drug-by-day interaction, as well as a significant drug main effect and day main effect, was of interest. Significant interactions were evaluated using analysis of simple effects to determine the days on which testosterone levels across the two treatment groups differed significantly at *p* < 0.05. A drug main effect required no follow-up analyses since there were only 2 levels of this factor, and the day main effect was not examined since this effect was addressed by the drug-by-day interaction term.

#### Cognitive and mood analyses

The data from each mood and cognitive variable also were analyzed in a 2-way, mixed factorial ANOVA in which the grouping factor “drug” consisted of the same 2 levels of treatment described above (i.e., testosterone and placebo); and the repeated-measure factor “day” consisted of 11 levels: day 13 (the pre-energy-deficit test day), days 15, 20, 22, 27, 29, 34, 36, and 41 (the energy-deficit testing days, with energy restriction and drug/placebo injections), day 54 (the midpoint of the post-laboratory time during which volunteers returned to pre-study diet/activity “free living”), and day 85 (the end of study day; Fig. 1). Degrees of freedom were corrected using the Greenhouse–Geisser procedure to adjust for potential sphericity violations across the day factor. Significant day main effects were followed-up with post hoc orthogonal linear, cubic, and quadratic contrasts rather than multiple pairwise comparisons in order to mitigate alpha inflation (running orthogonal contrasts required only 3 statistical comparisons, whereas running post hoc pairwise comparisons to examine changes among all possible combinations of 11 days would have required 55 statistical tests). In addition, orthogonal contrasts allowed us to assess overall patterns and relationships between levels of factors and response variables. Linear effects indicate a proportional change in the response variable across the factor levels, and cubic effects indicate any nonlinear patterns demonstrating the presence of more complex relationships. Quadratic effects capture the presence of U-shaped changes that could be present when comparing well-rested initial study days, high-stress testing days, and the stress recovery period during the free-living condition at the end of the study. The pattern of changes across the testing days was plotted for visual inspection using the auto-scaled Sparkline procedure available in Excel (Microsoft, Redmond, WA; Table 2). Sparklines provide graphical, auto-scaled plots of changes across testing days as an aid in the interpretation of statistically significant effects. For all statistical tests, *p* < 0.05 was used as the criterion for statistical significance. Effect sizes (eta-squared, *η*^2^) are provided in Table 1 for the behavioral variables.

**Table 1:** Statistical results from the ANOVAs conducted on phase 1, 2, and 3 testing days including *F* values, degrees of freedom, *P* values, and *η*^2^ (eta-squared)

Test	Measure	Drug/placebo main effect	Day of testing main effect	Drug × day interaction
Buss–Perry Aggression Questionnaire	Total	*F*(1,48) = 1.49, *p* = 0.23; *ή*^2^ = 0.030	*F*(3.2,152) = 0.85, *p* = 0.47; *ή*^2^ = 0.017	*F*(3.2,152) = 0.77, *p* = 0.52; *ή*^2^ = 0.016
	Physical	*F*(1,48) = 3.17, *p* = 0.08; *ή*^2^ = 0.062	*F*(3.4,164) = 0.56, *p* = 0.67; *ή*^2^ = 0.011	*F*(3.4,164) = 1.00, *p* = 0.40; *ή*^2^ = 0.020
	Verbal	*F*(1,48) = 0.14, *p* = 0.71; *ή*^2^ = 0.003	*F(4.4,213)* = *2.35, p* = *0.05; ή*^*2*^ = *0.047*	*F(*4.4,213) = 1.32, *p* = 0.26; *ή*^2^ = 0.027
	Hostility	*F*(1,48) = 2.49, *p* = 0.12; *ή*^2^ = 0.049	*F*(3.6,171) = 0.86, *p* = 0.48; *ή*^2^ = 0.018	*F*(3.6,171) = 0.76, *p* = 0.54; *ή*^2^ = 0.016
	Anger	*F*(1,48) = 0.04, *p* = 0.85; *ή*^2^ = 0.001	*F*(3.2,152) = 0.84, *p* = 0.48; *ή*^2^ = 0.017	*F*(3.2,152) = 0.76, *p* = 0.52; *ή*^2^ = 0.016
Profile of Mood States	TMD	*F*(1,47) = 0.14, *p* = 0.71; *ή*^2^ = 0.003	*F(6.0,280)* = *4.69, p* < *0.01; ή*^*2*^ = *0.091*	*F*(6.0,280) = 0.60, *p* = 0.73; *ή*^2^ = 0.013
	Tension	*F*(1,47) = 0.02, *p* = 0.88; *ή*^2^ = 0.000	*F*(5.8,271) = 2.02, *p* = 0.07; *ή*^2^ = 0.041	*F*(5.8,270) = 0.35, *p* = 0.90; *ή*^2^ = 0.007
	Depression	*F*(1,47) = 0.83, *p* = 0.37; *ή*^2^ = 0.017	*F*(5.1,237) = 2.17, *p* = 0.06; *ή*^2^ = 0.044	*F*(5.1,237) = 0.83, *p* = 0.53; *ή*^2^ = 0.017
	Anger	*F*(1,47) = 0.003, *p* = 0.96; *ή*^2^ = 0.000	*F(5.8,272)* = *3.04, p* = *0.01; ή*^*2*^ = *0.061*	*F*(5.8,272) = 0.54, *p* = 0.77; *ή*^2^ = 0.011
	Vigor	*F*(1,47) = 0.41, *p* = 0.52; *ή*^2^ = 0.009	*F*(5.5,259) = 1.12, *p* = 0.35; *ή*^2^ = 0.023	*F*(5.5,259) = 1.18, *p* = 0.32; *ή*^2^ = 0.024
	Fatigue	*F*(1,47) = 0.65, *p* = 0.43; *ή*^2^ = 0.014	*F(6.6,310)* = *13.76, p* < *0.01;ή*^*2*^ = *0.227*	*F*(6.6,310) = 0.40, *p* = 0.90; *ή*^2^ = 0.008
	Confusion	*F*(1,47) = 0.07, *p* = 0.80; *ή*^2^ = 0.001	*F(6.2,290)* = *2.32, p* = *0.03; ή*^*2*^ = *0.047*	*F*(6.2,290) = 0.61, *p* = 0.73; *ή*^2^ = 0.013
Balloon Analog Risk Task	N Pop 8	*F*(1,48) = 0.41, *p* = 0.53; *ή*^2^ = 0.018	*F(6.2,296)* = *2.74, p* = *0.01; ή*^*2*^ = *0.054*	*F*(6.2,296) = 1.66, *p* = 0.13; *ή*^2^ = 0.033
	N Pop 32	*F*(1,48) = 0.06, *p* = 0.81; *ή*^2^ = 0.001	*F(5.1,247)* = *2.67, p* = *0.02; ή*^*2*^ = *0.053*	*F*(5.1,247) = 1.21, *p* = 0.31; *ή*^2^ = 0.025
	N Pop 128	*F*(1,48) = 0.28, *p* = 0.60; *ή*^2^ = 0.006	*F*(5.1,246) = 1.72, *p* = 0.13; *ή*^2^ = 0.035	*F*(5.1,246) = 1.09, *p* = 0.37; *ή*^2^ = 0.022
Ultimatum Game	Total Coll	*F*(1,48) = 0.33, *p* = 0.57; *ή*^2^ = 0.007	*F*(6.7,322) = 1.70, *p* = 0.11; *ή*^2^ = 0.034	*F*(6.7,322) = 1.87, *p* = 0.08; ή^2^ = 0.037
	N accepted	*F*(1,48) = 0.07, *p* = 0.79; *ή*^2^ = 0.001	*F(4.4,212)* = *2.54, p* = *0.04; ή*^*2*^ = *0.050*	*F*(4.4,212) = 0.99, *p* = 0.42; *ή*^2^ = 0.020
	N rejected	*F*(1,48) = 0.08, *p* = 0.78; *ή*^2^ = 0.002	*F(4.6,222)* = *2.32, p* = *0.05; ή*^*2*^ = *0.046*	*F*(4.6,222) = 0.89, *p* = 0.48; *ή*^2^ = 0.018
	Time outs	*F*(1,48) = 0.20, *p* = 0.66; *ή*^2^ = 0.004	*F*(2.1,101) = 1.45, *p* = 0.24; *ή*^2^ = 0.029	*F*(2.1,101) = 0.72, *p* = 0.50; *ή*^2^ = 0.015
	RT accepted	*F*(1,48) = 2.20, *p* = 0.15; *ή*^2^ = 0.002	*F(6.3,301)* = *3.78, p* < *0.01; ή*^*2*^ = *0.024*	*F*(6.3,301) = 1.35, *p* = 0.24; *ή*^2^ = 0.019
	RT rejected	*F*(1,24) = 2.21, *p* = 0.15; *ή*^2^ = 0.044	*F*(5.1,122) = 1.40, *p* = 0.23; *ή*^2^ = 0.073	*F*(5.1,122) = 1.10, *p* = 0.36; *ή*^2^ = 0.027
Reading Mind in the Eyes	Time outs	*F(1,48)* = *5.51, p* = *0.02; ή*^*2*^ = *0.103*	*F*(5.7,275) = 0.98, *p* = 0.44; *ή*^2^ = 0.020	*F*(5.7,275) = 0.87, *p* = 0.51; *ή*^2^ = 0.018
	Correct	*F*(1,48) = 1.69, *p* = 0.20; *ή*^2^ = 0.034	*F(4.7,224)* = *4.94, p* < *0.01; ή*^*2*^ = *0.093*	*F*(4.7,224) = 0.71, *p* = 0.61; *ή*^2^ = 0.015
	Incorrect	*F*(1,48) = 1.80, *p* = 0.19; *ή*^2^ = 0.034	*F(4.6,222)* = *5.15, p* < *0.01; ή*^*2*^ = *0.097*	*F*(4.6,222) = 0.64, *p* = 0.65; *ή*^2^ = 0.013
	RT	*F(1,48)* = *6.44, p* = *0.01; ή*^*2*^ = *0.118*	*F(6.4,307)* = *14.91, p* < *0.01; ή*^*2*^ = *0.237*	*F*(6.4,307) = 0.80, *p* = 0.58; *ή*^2^ = 0.016
Scanning Visual Vigilance	Fals alarm	*F*(1,47) = 0.37, *p* = 0.55; *ή*^2^ = 0.008	*F*(3.0,143) = 0.80, *p* = 0.50; *ή*^2^ = 0.017	*F*(3.0,143) = 1.30, *p* = 0.28; *ή*^2^ = 0.027
	Corr Resp	*F*(1,47) = 0.06, *p* = 0.81; *ή*^2^ = 0.001	*F(6.2,292)* = *3.51, p* < *0.01; ή*^*2*^ = *0.070*	*F*(6.2,292) = 0.59, *p* = 0.75; *ή*^2^ = 0.012
Psychomotor Vigilance	Prem Resp	*F*(1,47) = 0.33, *p* = 0.57; *ή*^2^ = 0.007	*F*(4.6,216) = 0.95, *p* = 0.45; *ή*^2^ = 0.020	*F*(4.6,216) = 0.69, *p* = 0.62; *ή*^2^ = 0.014
	Time outs	*F*(1,47) = 0.31, *p* = 0.58; *ή*^2^ = 0.007	*F(5.6,265)* = *3.17, p* < *0.01; ή*^*2*^ = *0.063*	*F*(5.6,265) = 0.69, *p* = 0.65; *ή*^2^ = 0.014
	Corr Resp	*F*(1,47) = 0.18, *p* = 0.67; *ή*^2^ = 0.004	*F(5.9,278)* = *3.00, p* = *0.01; ή*^*2*^ = *0.060*	*F*(5.9,278) = 0.76, *p* = 0.60; *ή*^2^ = 0.016
	RT	*F*(1,44) = 0.10, *p* = 0.75; *ή*^2^ = 0.002	*F(7.2,318)* = *4.55, p* < *.0.01; ή*^*2*^ = *0.094*	*F*(7.2,318) = 0.70, *p* = 0.68; *ή*^2^ = 0.012
Matching to Sample	Corr Resp	*F*(1,48) = 0.32, *p* = 0.58; *ή*^2^ = 0.007	*F*(7.5,360) = 1.48, *p* = 0.17; *ή*^2^ = 0.030	*F*(7.5,360) = 0.99, *p* = 0.44; *ή*^2^ = 0.020
	Time outs	*F*(1,48) = 0.13, *p* = 0.72; *ή*^2^ = 0.003	*F*(6.1,292) = 1.17, *p* = 0.32; *ή*^2^ = 0.024	*F*(6.1,292) = 1.25, *p* = 0.28; *ή*^2^ = 0.025
	RT	*F*(1,48) = 0.37, *p* = 0.55; *ή*^2^ = 0.008	*F(5.5,265)* = *11.57, p* < *0.01; ή*^*2*^ = *0.194*	*F*(5.5,265) = 2.01, *p* = 0.07; *ή*^2^ = 0.040
Grammatical Reasoning	Corr Resp	*F*(1,48) = 0.41, *p* = 0.52; *ή*^2^ = 0.009	*F(6.2,299)* = *2.40, p* = *0.03; ή*^*2*^ = *0.048*	*F*(6.2,299) = 0.66, *p* = 0.69; *ή*^2^ = 0.013
	Incorr Resp	*F*(1,48) = 0.53, *p* = 0.47; *ή*^2^ = 0.011	*F(5.5,263)* = *2.48, p* = *0.03; ή*^*2*^ = *0.049*	*F*(5.5,263) = 0.50, *p* = 0.79; *ή*^2^ = 0.010
	Time outs	*F*(1,48) = 1.03, *p* = 0.31; *ή*^2^ = 0.021	*F(3.8,180)* = *3.75, p* < *0.01; ή*^*2*^ = *0.072*	*F*(3.8,180) = 0.47, *p* = 0.76; *ή*^2^ = 0.009
	RT	*F*(1,48) = 3.34, *p* = 0.07; *ή*^2^ = 0.065	*F(4.6,221)* = *7.99, p* < *0.01; ή*^*2*^ = *0.143*	*F*(4.6,221) = 0.43, *p* = 0.81; *ή*^2^ = 0.009
N-Back-1	Overall Corr	*F*(1,48) = 0.08, *p* = 0.78; *ή*^2^ = 0.002	*F*(4.4,211) = 1.13, *p* = 0.34; *ή*^2^ = 0.023	*F*(4.4,211) = 0.43, *p* = 0.81; *ή*^2^ = 0.009
	Correct	*F*(1,48) = 0.10, *p* = 0.75; *ή*^2^ = 0.002	*F*(6.8,327) = 0.92, *p* = 0.49; *ή*^2^ = 0.019	*F*(6.8,327) = 0.69, *p* = 0.68; *ή*^2^ = 0.014
	False Pos	*F*(1,48) = 0.25, *p* = 0.62; *ή*^2^ = 0.005	*F*(2.1,103) = 1.07, *p* = 0.35; *ή*^2^ = 0.022	*F*(2.1,103) = 0.58, *p* = 0.57; *ή*^2^ = 0.012
	RT	*F*(1,41) = 0.28, *p* = 0.60; *ή*^2^ = 0.007	*F(6.7,275)* = *2.56, p* = *0.02; ή*^*2*^ = *0.059*	*F*(6.7,275) = 1.76, *p* = 0.10; *ή*^2^ = 0.041
N-Back-2	Overall Corr	*F*(1,48) = 0.12, *p* = 0.73; *ή*^2^ = 0.002	*F*(5.4,257) = 1.00, *p* = 0.42; *ή*^2^ = 0.020	*F*(5.4,257) = 0.89, *p* = 0.50; *ή*^2^ = 0.018
	Correct	*F*(1,48) = 0.57, *p* = 0.45; *ή*^2^ = 0.012	*F(7.7,372)* = *2.25, p* = *0.03; ή*^*2*^ = *0.045*	*F*(7.7,372) = 0.47, *p* = 0.87; *ή*^2^ = 0.010
	False Pos	*F*(1,48) = 0.01, *p* = 0.93; *ή*^2^ = 0.000	*F*(2.9,140) = 0.64, *p* = 0.59; *ή*^2^ = 0.013	*F*(2.9,140) = 1.05, *p* = 0.37; *ή*^2^ = 0.021
	RT	*F*(1,43) = 0.08, *p* = 0.78; *ή*^2^ = 0.002	*F*(6.5,281) = 1.22, *p* = 0.30; *ή*^2^ = 0.027	*F*(6.5,281) = 0.77, *p* = 0.60; *ή*^2^ = 0.018

Prior to analyzing the behavioral data using parametric procedures, we evaluated non-parametric statistical approaches since behavioral data frequently are not normally distributed. However, the design of this study does not lend itself to a non-parametric approach. It is well-documented that ANOVA is tolerant to violation of normality assumptions and that, in many circumstances, ANOVA is preferable to non-parametric procedures (for example, see Knief and Forstmeier 2021; Blanca et al. 2023).

Very few data points were missing, on rare occasion when this occurred for individual behavioral test variables, that volunteer’s data were dropped for analysis due to the limitations of the ANOVA procedure. Missing data were not estimated. In no case was it necessary to drop more than one volunteer’s individual behavioral test data. Overall, data for 3 behavioral tests for one volunteer were not available due to a computer malfunction.

## Results

### Testosterone levels

**Fig. 2:**
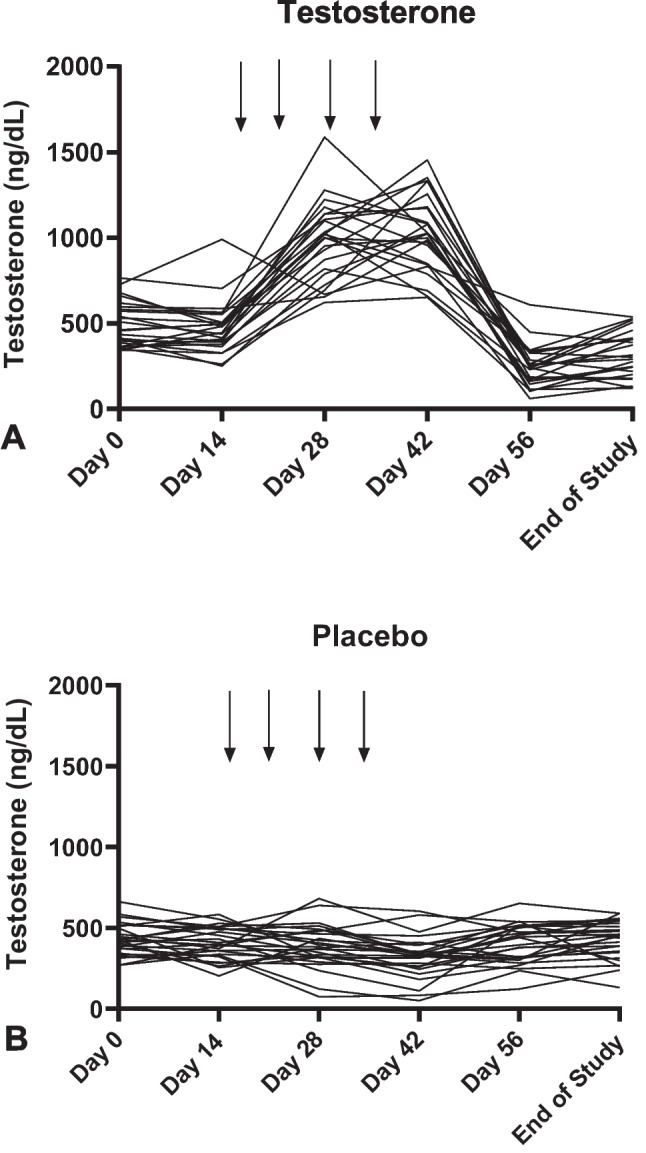
Plots of individual levels of testosterone in all 3 phases of the study. **A** The data from volunteers who received testosterone. **B** The data from those who received placebo. Arrows indicate days when fasted blood samples were drawn (days 0, 14, 28, 42, 56, and end of study)

**Fig. 3:**
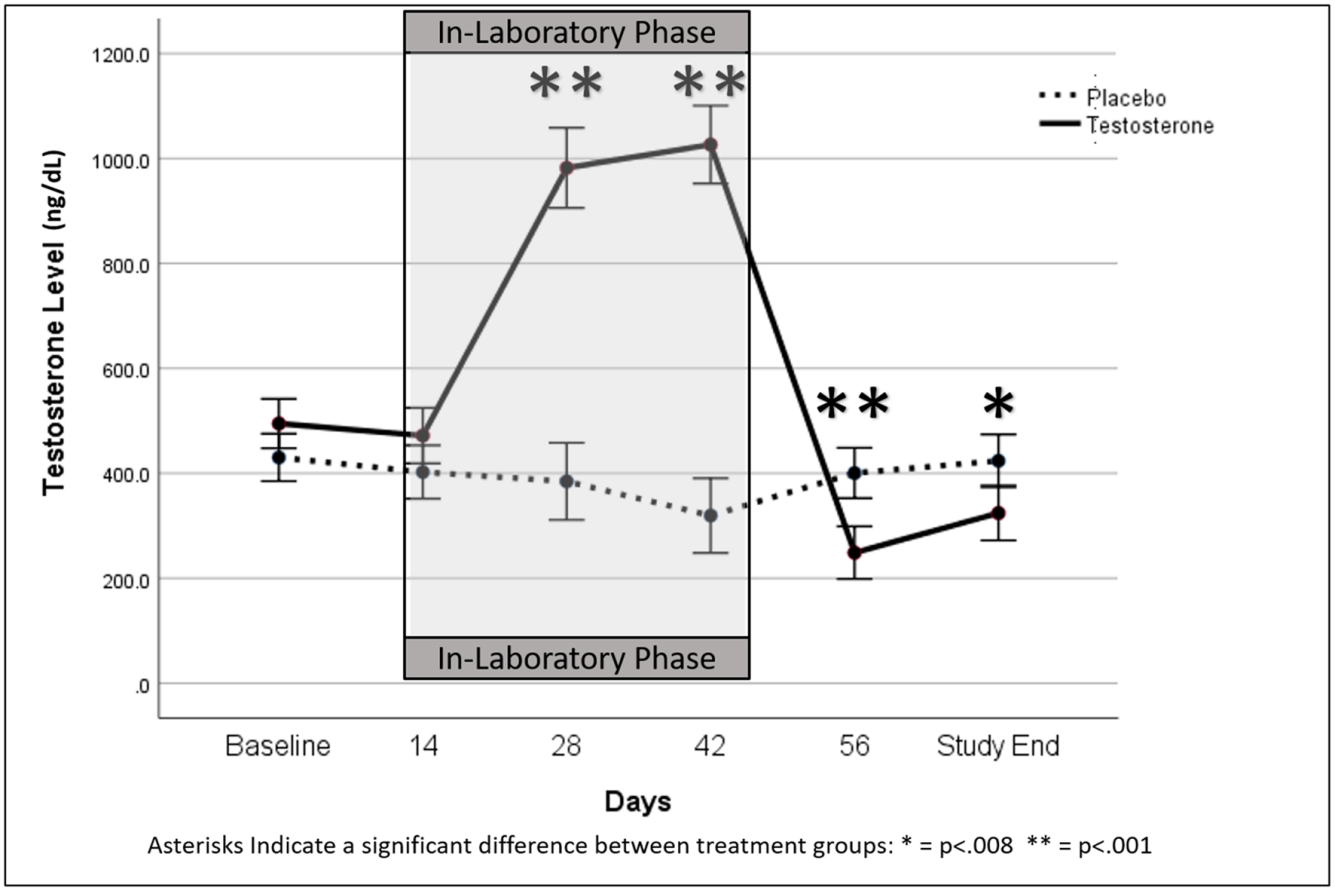
Serum testosterone levels on the days when fasted blood samples were drawn (days 0, 14, 28, 42, 56, and end of study); asterisks indicate the days on which there were statistically significant differences between the testosterone and placebo treatment groups (*p = .008; **p < .001); although significance is not indicated, there were significant differences in testosterone levels between day 0 and day 42 of the placebo group (*p* < .05)

Mean testosterone levels over time are provided in Table 2, and plots of individual data are provided in Fig. 2. Levels of testosterone in a number of volunteers who received testosterone exceeded what would be considered normal levels (300–1001 ng/dL) of the hormone in young, healthy males (Fig. 2A). A significant drug-by-day interaction (*F*[3.12,150] = 115.47; *p* < 0.001; *η*^2^ = 0.71) and a significant drug main effect (*F*[1,48] = 51.93; *p* < 0.001; *η*^2^ = 0.96) were observed on testosterone serum levels. In addition, there was a significant day main effect (*F*[3.12,150] = 80.13; *p* < 0.001; *η*^2^ = 0.63). The magnitude of these effect sizes (*η*^2^) indicate that the effects are quite large based on standard criteria (Murphy and Myors 2004). As expected, for the placebo treatment condition, testosterone levels were lower overall compared to those in the testosterone treatment condition due to the combined effects of energy deprivation and substantial aerobic exercise. In terms of the interaction between treatment and day, post hoc analyses indicated higher testosterone in the drug vs. placebo group on day 0 (*F*[1,48] = 3.97; *p* = 0.052; *η*^2^ = 0.076), day 28 (*F*[1,48] = 128.37; *p* < 0.001; *η*^2^ = 0.73), and day 42 (*F*[1,48] = 190.67; *p* < 0.001; *η*^2^ = 0.80). This was followed by lower testosterone in the drug vs. placebo group on day 56 (*F*[1,48] = 19.31; *p* < 0.001; *η*^2^ = 0.29) and the last day of the study (*F*[1,48] = 7.64; *p* = 0.008; *η*^2^ = 0.14) (Fig. 3).

**Table 2:**
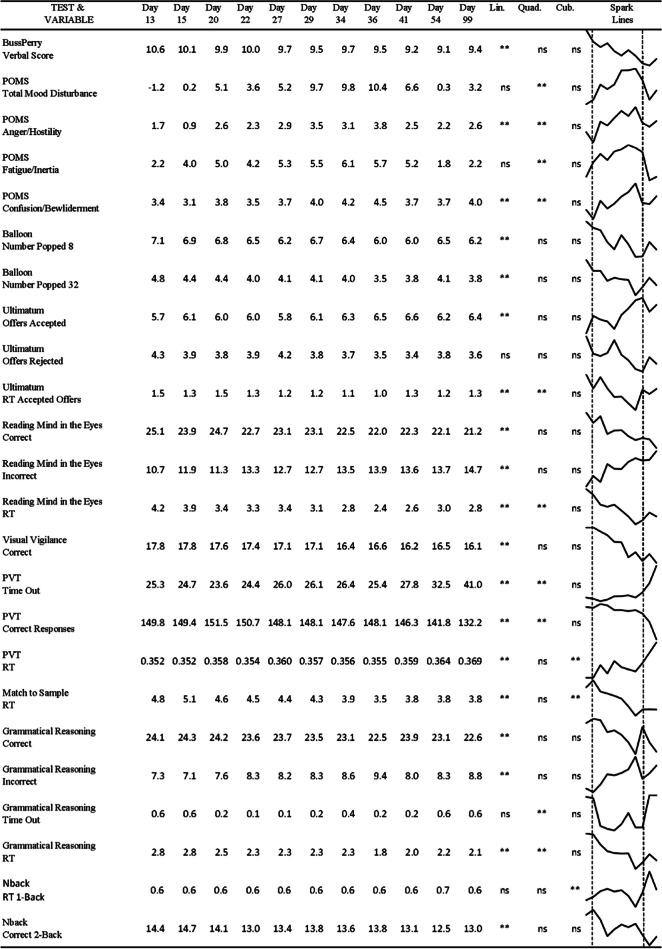
Day main effects across phases 1, 2, and 3. Significant contrasts and Excel Sparkline plots (with test phases delimited by dashed line) included

### Behavioral tests

There were no significant interactions between the drug and day factors, indicating that there were no differences across test days on cognition, emotion, or behavior as a result of whether volunteers received placebo versus testosterone (Table 1). There was a main effect of drug (testosterone vs. placebo) on the reaction time measure and on the time out measure for Reading the Mind in the Eyes. There were longer response times and more time-outs in the placebo versus testosterone groups. There were trends for behavioral effects on the physical subscale of the Buss–Perry Aggression Questionnaire (*F*(1,48) = 3.17, *p* = 0.08; *η*^2^ = 0.062) as well as reaction time for Grammatical Reasoning (*F*(1,48) = 3.34, *p* = 0.07; *η*^2^ = 0.065).

There were 24 significant day main effects indicative of changes in cognition and mood across the 11 test days of the study (days 13–85). The statistical significance/non-significance of these contrasts along with mean changes throughout the study, and sparklines to illustrate the pattern of change, is presented in Table 2. Many of these effects would be classified as “medium” or “large” (Murphy and Myors 2004).

Of the 24 measures on which there were day main effects, significant linear trends were observed on most measures with a largely consistent pattern of degraded performance and mood over time. The number of correct responses on the PVT decreased over time (*F*[1,47] = 13.32; *p* = 0.001; *η*^2^ = 0.221) while reaction time (*F*[1,44] = 14.72; *p* < 0.001;*η*^2^ = 0.251) and number of time outs (*F*[1,47] = 12.74; *p* = 0.001; *η*^2^ = 0.213), indicating loss of attention, on this widely accepted test of reaction time and vigilance increased. Correct responses on Grammatical Reasoning (*F*[1,48] = 5.05; *p* = 0.029; *η*^2^ = 0.095), the 2-back task (*F*[1,48] = 9.30; *p* = 0.004; *η*^2^ = 0.162), and Visual Vigilance (*F*[1,47] = 31.91; *p* < 0.001; *η*^2^ = 0.404) also decreased. There were several measures that appeared to improve, including faster reaction times on Grammatical Reasoning (*F*[1,48] = 15.30; *p* < 0.001; *η*^2^ = 0.242) and Matching to Sample (*F*[1,48] = 37.70; *p* < 0.001; *η*^2^ = 0.440), and fewer balloon pops on both the 8-maximum (*F*[1,48] = 6.56; *p* = 0.014; *η*^2^ = 0.120) and 32-maximum (*F*[1,48] = 4.66; *p* = 0.036; *η*^2^ = 0.088) BART, perhaps reflecting a learning effect.

With regard to mood/psychological effects, two subscales on the POMS substantially worsened over time, Confusion/Bewilderment (*F*[1,47] = 5.40; *p* = 0.025; *η*^2^ = 0.103) and Anger/Hostility (*F*[1,47] = 6.55; *p* = 0.014; *η*^2^ = 0.122). In addition, the ability to read the mind in the eyes, a measure of the ability to recognize the thoughts and feelings of others, also linearly deteriorated across the testing days (*F*[1,48] = 14.15; *p* < 0.001; *η*^2^ = 0.228), although the reaction time on this test improved (*F*[1,48] = 52.06; *p* < 0.001; *η*^2^ = 0.520). The presence of a significant linear trend indicates a general, collective upward or downward change across the entire study, not necessarily across every pair of testing days.

In addition to the linear effects, there were also quadratic (curvilinear) effects on 10 out of the 25 measures on which overall day effects were observed. As can be seen in Table 2, the linear decline on PVT performance continued upon transition to the free-living phase (*F*[1,47] = 6.98; *p* = 0.011; *η*^2^ = 0.129) and the improvement in reaction times on the Ultimatum Game (*F*[1,48] = 27.08; *p* < 0.001; *η*^2^ = 0.361), Reading the Mind in the Eyes Test (*F*[1,48] = 15.59; *p* < 0.001; *η*^2^ = 0.245), and Grammatical Reasoning task (*F*[1,48] = 10.14; *p* = 0.003; *η*^2^ = 0.174) observed during the in-house testing phase was partially reversed during the free-living phase. The declines in POMS Total Mood Disturbance (*F*[1,47] = 15.53; *p* < 0.001; *η*^2^ = 0.248), Anger/Hostility (*F*[1,47] = 7.95; *p* = 0.007; *η*^2^ = 0.145), Fatigue/Inertia (*F*[1,47] = 59.36; *p* < 0.001; *η*^2^ = 0.558), and Confusion/Bewilderment (*F*[1,47] = 4.73; *p* = 0.035; *η*^2^ = 0.091) observed during the in-house testing phase reversed upon release from the laboratory environment. There were only a few significant cubic effects (3 of the 25; Table 2).

## Discussion

This study found cognitive functions sometimes reported to be associated with exogenous testosterone treatment including aggression, risk-taking, competition, social cognition, and mood, as well as other functions such as vigilance, were not affected by chronic, repeated administration of testosterone enanthate (200 mg/week) for 4 weeks during the unique circumstances of this study—chronic administration of testosterone and a severe energy deficit in healthy male volunteers. This study was designed to simulate the energy deficit that typically occurs during sustained military operations. As expected, there were significant reductions in endogenous levels of testosterone in placebo-treated volunteers due to caloric deprivation. Also, as anticipated from prior research, cognitive function was compromised by substantial, sustained energy deprivation (Keys et al.^1950^). The negative findings of this study are specific to males experiencing a severe energy deficit. They should not be generalized to other formulations, doses, or acute administration of testosterone or the effects of endogenous levels of the hormone in humans or other animals. In addition, as the study was designed to detect a change in muscle mass, not behavioral changes, a study with a larger *N* or using more sensitive behavioral tasks may have found effects of testosterone as many have (e.g., O’Connor 2007; Dreher et al. 2016; Nave et al. 2017; Bird et al. 2019; Geniole et al. 2019; Kutlikova et al. 2023; Vartanian et al. 2023).

Regular assessment of serum testosterone demonstrated testosterone-treated volunteers experienced increasing levels of testosterone during the treatment phase of the study which peaked after the final testosterone injection and then fell to below baseline levels, a typical observation following cessation of treatment with testosterone. Testosterone levels are regulated by a negative feedback loop, so that when high levels are present, its secretion is inhibited. Therefore, when exogenous testosterone is administered, endogenous testosterone synthesis decreases and takes some time to recover if exogenous testosterone administration is abruptly halted, as occurred in this study on day 56 and the end-of-study (Handelsman et al. 2022).

The lack of effects of testosterone enanthate (200 mg/week) for 4 weeks on cognitive function observed here are in agreement with a recent review paper on the behavioral effects of testosterone (Yalamanchi and Dobs 2017). However, it should be noted in another arm of this study that it was found that testosterone increased sensitivity to negative feedback if volunteers received negative feedback first (Vartanian et al. 2023). Yalamanchi and Dobs (2017) examined the literature on exogenous testosterone treatment and concluded that “Overall, available data are not suggestive of a clear benefit of testosterone supplementation in multiple domains of cognition and in mood” (Yalamanchi and Dobs 2017, page 525). In addition, Buskbjerg et al. (2019) conducted a meta-analysis of 21 controlled trials and concluded the available data did not support the hypothesis that chronic administration of testosterone had beneficial effects on cognitive function of men with normal testosterone levels. They concluded that testosterone should not be administered to improve cognition in eugonadal men, especially given its potential for adverse effects including possible chronic adverse effects.

This study was unique as healthy volunteers, whose testosterone levels were lowered by energy deprivation, were treated with exogenous testosterone to replace their own testosterone. If treatment with testosterone was not administered, those volunteers would have had low levels of endogenous testosterone as demonstrated by the very low testosterone levels of the control volunteers. The results of this study therefore indicate that exogenous, slow-release testosterone, in the dose (200 mg/week for 4 weeks) and specific formulation (testosterone enanthate) used here, does not improve or degrade certain aspects of cognition even when endogenous levels are temporarily suppressed. However, this study should not be interpreted as suggesting testosterone does not affect behavior. For example, studies that have found effects of testosterone on behavior include Dreher et al. (2016), Nave et al. (2017), Bird et al. (2019), Geniole et al. (2019), and Kutlikova et al. (2023).

It is important to differentiate the effects of exogenous from endogenous testosterone. The lack of effects of exogenous testosterone on cognitive function in the men in the present study does not contradict reports of associations between endogenous testosterone and behavior in humans or other animals or acute administration. Many years of exposure to endogenous testosterone in males, including during puberty when the brain is undergoing substantial growth and rewiring, undoubtedly have different effects on the central nervous system than several weeks of treatment with exogenous testosterone (Sisk and Zehr 2005). Similarly, acute vs. chronic administration of testosterone can be expected to have different effects on testosterone levels and behavior. Acute studies of testosterone often result in much higher plasma levels of testosterone than chronic studies (Liu et al. 2003).

The absence of cognitive and mood changes in our study is in agreement with Gillam (2018) who concluded men with symptomatic hypogonadism are unlikely to experience improvements in energy, mood, or cognition as a result of testosterone therapy. Behavioral claims made by advertisers of so-called testosterone “boosters,” sold as dietary supplements, should be regarded with skepticism given the findings of this study to others (Kovac et al. 2016; Balasubramanian et al. 2019). These dietary supplements have not been shown to increase testosterone or be safe. Overall, the results of this arm of the trial suggest that testosterone, if used in military operations where testosterone levels are lowered by exposure to severe energy deprivation, will have no adverse cognitive effects.

This study conducted comprehensive physical performance and body composition measurements to determine if testosterone can mitigate the adverse effects of sustained military operations on these factors (Pasiakos et al. 2019). Weekly doses of 200 mg doses of testosterone enanthate significantly reduced loss of lean body mass during the 4-week period of severe energy deficit (Pasiakos et al. 2019) and affected other parameters such as protein synthesis and androgen receptor expression (Howard et al. 2020; 2022). However, testosterone treatment did not affect physical performance assessed using isometric and isokinetic knee extension tests nor did it adversely affect multiple health-related biomarkers and had no detectable adverse effects on cognitive function, although the study was not designed to examine behavior (Pasiakos et al. 2019). The absence of adverse effects of testosterone on cognitive function and its positive effects on lean body mass suggest that exogenous testosterone administration may be a viable approach to sustaining lean body mass during sustained military operations. However, additional studies using different formulations and/or doses of testosterone should be conducted, for example, with the formulations of testosterone that have longer half-lives than testosterone enanthate.

## Limitations

The findings of this study are of limited generalizability. It was not designed to examine behavioral variables but was a secondary arm of a study designed to evaluate the effects of chronic administration of testosterone on muscle mass and physical performance. As such, it was not designed based on power estimates of the behavioral parameters assessed and may have been underpowered. Many of the studies of effects of acute, exogenous testosterone administration that have found effects have much larger sample sizes, often in the hundreds (Dreher et al. 2016; Nave et al. 2017; Bird et al. 2019; Geniole et al. 2019; Kutlikova et al. 2023). However, it should be noted in this study that the behavioral tests were repeatedly administered to the volunteers during testosterone treatment, a study strength which compensates, in part, for the relatively small sample size. One another possible limitation of this study is that the dose of testosterone used may not have been sufficient to result in behavioral effects as a number of studies have found effects using much larger doses (O’Connor 2007). There is substantial variation in testosterone doses, formulation (immediate or sustained release), gender, the genetics and personality of subjects, and duration of treatment in studies with behavioral end points; accordingly, it is difficult to draw conclusions from the literature.

Although difficult and costly to conduct, future investigators should consider conducting dose–response and other parametric studies as well as distinguishing between acute and chronic effects using identical behavioral tasks. Since acute studies have reported personality characteristics and genetic factors can affect response to testosterone, these variables should be accounted for in future studies (Bird et al. 2019; Geniole et al. 2019; Losecaat Vermeer et al. 2020). One additional limitation was that only a certain number of behaviors could be assessed, and therefore, effects on functions we did not examine would not be detected. However, we attempted to select a number of functions that were likely to be altered by testosterone administration. Furthermore, even seemingly small differences in specific cognitive tests can result in different findings and the psychometric properties of the tasks used may have resulted in ceiling or floor effects. In that regard, it should be noted that we were unable to analyze the ultimatum task with “size of offer” as an individual variable as is done in many studies. Also, it should be noted that levels of testosterone in the placebo group could have been affected by participation in behavioral testing. For example, performance on the Ultimatum Game can affect testosterone levels depending on whether the volunteer wins or loses (Carré et al 2013; Archer 2006). Additionally, our study sample was relatively young, and although we successfully created a testosterone deficiency in them, the results could have been different if elderly men with lower endogenous levels of testosterone were studied.

Finally, none of the volunteers were active duty military personnel, and selection of volunteers from the civilian population may have resulted in inclusion of individuals with somewhat different personality traits than the general population due to their willingness to comply with the extensive study requests such as refraining from medication, alcohol, nicotine, and caffeine use during the entire study period. Several personality traits, such as self-control, can reduce the effects of testosterone on aggression (Carré et al. 2017; Geniole et al. 2019).

## Conclusions

In summary, in this double-blind, placebo-controlled trial of 200 mg testosterone enanthate administered weekly for 4 weeks, during a period of severe energy restriction designed to simulate military field operations, significant changes in cognition or mood in young healthy males were not detected. Among the functions examined were behaviors believed to be related to endogenous and exogenous testosterone levels, such as aggression, generosity, anger, and risk-taking. However, as noted above, there were many limitations to this study as it was conducted as a secondary arm of a study designed to examine the effects of chronic testosterone on muscle mass and physical performance including its relatively small sample size compared to many studies of acute administration of testosterone. The results of this study should not be considered as contradicting studies of acute administration of testosterone and also should not be generalized to the effects of naturally present, endogenous testosterone in humans or other species. This study does indicate that testosterone treatment, in the dose, formulation, and duration we used, does not have adverse behavioral effects.

## Data Availability

De-identified data are available upon request if approved by the IRB.

## Declarations

### Disclaimer

The views expressed in this paper are those of the authors and do not reflect the official policy of the Department of Army, Department of Defense, National Institutes of Health, Department of Health and Human Services, or U.S. Government. Any citations of commercial organizations and trade names in this report do not constitute an official Department of the Army or Health and Human Services endorsement or approval of the products or services of these organizations.
